# Chemical Gas Telemetry System Based on Multispectral Infrared Imaging

**DOI:** 10.3390/toxics11010083

**Published:** 2023-01-15

**Authors:** Kun Li, Shaoli Duan, Lingling Pang, Weilai Li, Zhixiong Yang, Yaohang Hu, Chunchao Yu

**Affiliations:** 1 Kunming Institute of Physics, Kunming 650223, China; 2 School of Opto-Electronical Engineering, Xi’an Technological University, Xi’an 710021, China; 3 College of Physics and Electronic Information, Yunnan Normal University, Kunming 650029, China

**Keywords:** gas detection, infrared imaging, multispectral

## Abstract

Environmental monitoring, public safety, safe production, and other areas all benefit greatly from the use of gas detection technologies. The infrared image of a gas could be used to determine its type from a long distance in gas detection. The infrared image can show the spatial distribution of the gas cloud and the background, allowing for long-distance and non-contact detection during safety production and hazardous chemical accident rescue. In this study, a gas detection system based on multispectral infrared imaging is devised, which can detect a variety of gases and determine the types of gas by separating the infrared radiation. It is made up of an imaging optical system, an uncooled focal plane detector, a filter controller, and a data gathering and processing system. The resolution of the infrared image is 640 × 512 and the working band of the system is 6.5~15 μm. The system can detect traces of pollutants in ambient air or gas clouds at higher concentrations. Ammonia, sulfur hexafluoride, methane, sulfur dioxide, and dimethyl methyl phosphonate were all successfully detected in real time. Ammonia clouds could be detected at a distance of 1124.5 m.

## 1. Introduction

Chemical gases are used more and more widely in industrial production and daily life. For example, ammonia is used as raw material in industrial production, and natural gas is used as energy in daily life. The leakage of chemical gas not only harms the environment but also may cause serious accidents. Rapid gas detection can detect potential safety hazards early in the production, storage, and transportation of chemical gases. It is feasible to avoid deaths and property damage by precisely pinpointing the gas leakage site as well as the dispersion and diffusion trend of the released gas in space [1,2,3]. Electrochemical detection, catalytic combustion, and laser detection are the most common methods of detecting gas [4]. The electrochemical approach is easily influenced by other gases, since it depends on the electrical signal produced by the chemical interaction between the target gas and the sensor electrode. Heat is created by the catalytic combustion process. The resistance of the detection electrode wire rises as the temperature rises. It is typically used to detect flammable gases. Laser detection is based on the selective absorption of light by gas molecules. Only one gas can be detected at one laser wavelength. To identify the kind and concentration of the target gas at a long distance, infrared imaging gas detection uses images of gas on the infrared spectrum.

It can show the spatial distribution and background of gas clouds. Infrared imaging gas detection technology has developed quickly due to its advantages of high detection efficiency, long detection distance, dynamic and intuitive images, and quick discovery of leakage sources [5,6,7,8,9,10]. Hyperspectral infrared imaging gas detection can accurately identify the type of a gas, and has the ability to detect the composition of a mixed gas [11,12,13,14,15]. However, hyperspectral infrared imaging gas detection systems are complex and require huge amounts of data processing. The multispectral infrared imaging gas detection system has a simple structure and can meet the needs of most places. It only requires one spectral band to image a specific gas using infrared technology. The absorption spectral band only spans a few tens or hundreds of nanometers, which only makes up a small fraction of the entire infrared spectrum. The filter can be used to select a specific spectral band. A narrow-band filter can perform infrared imaging of a gas with a particular absorption peak, but the detector only receives a limited quantity of infrared light energy. Adjacent long pass filters can also identify a certain gas. The detector receives a large amount of infrared radiation energy, which improves the signal-to-noise ratio of the infrared image. The multispectral infrared imaging gas detection system uses a number of filters to separate the object’s infrared radiation. The second sight system produced by Bertin is equipped with a motorized filter wheel with six positions, one for each filter. The second sight system is a versatile sensing instrument capable of detecting a variety of combustible and industrial gases [16]. Telops has developed dynamic multispectral imaging systems which allow synchronized acquisition on eight channels at a high frame rate, using a motorized filter wheel. [17]. A wide-band gas leak imaging detection system using uncooled focal plane array (UFPA) is proposed by Jin et al. for the quick detection of gas leaks. This system can image gases in the wave range of 3 μm~12 μm. By changing the sub-band filters, different gases can be detected with high sensitivity [18]. Although Bertin has done a lot of work in multispectral infrared imaging gas detection, they have not clearly introduced the gas detection method. In this paper, a multispectral infrared imaging gas detection system was built using six long-pass and short-pass infrared filters. An infrared camera with a working band of 3 μm~15 μm was used. The acquired infrared images were processed in real time and the filter wheel rotates at a constant speed. The correlation coefficient algorithm of the spectrum could identify the type and the distribution of gas clouds with few false positives. Real-time detection of ammonia (NH_3_), sulfur hexafluoride (SF_6_), methane (CH_4_), sulfur dioxide (SO_2_), and dimethyl methyl phosphonate (DMMP) has been accomplished.

## 2. Experimental Equipment and Methods

### 2.1. Camera and Detection Principle

**Figure 1:**
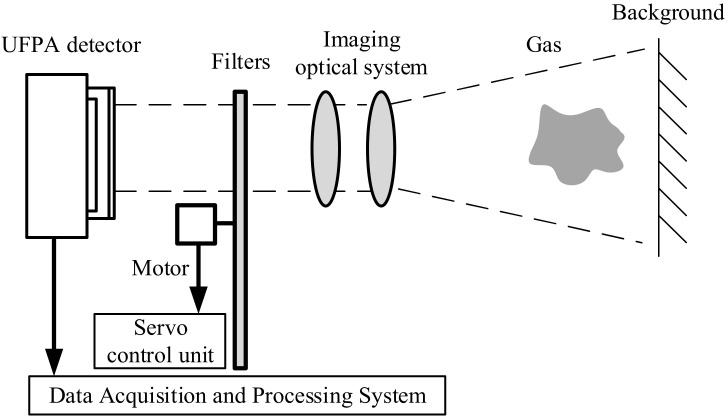
Composition of multispectral infrared imaging gas detection system.

Infrared imaging gas detection is based on the absorption of infrared radiation by gas molecules at specified wavelengths. The difference in the infrared image between the gas cloud and the background is caused by the absorption of infrared radiation by the gas. As a result, the area of gas diffusion can be located. A specific temperature difference between the gas and the background is needed to produce a gas image with obvious contrast. The temperature difference is usually determined by the gas concentration and the sensitivity of the infrared camera. The expansion of gas volume will cause a decrease in temperature during imaging detection of leaking high-pressure gas. As a result, the temperature difference between the gas and the surroundings is substantial. Industrial exhaust gases released from chimneys have a high temperature. The temperature difference between the gas and the sky is also significant. As shown in Figure 1, the multispectral infrared imaging gas detection system is mainly composed of an uncooled focal plane detector, filters, an imaging optical system, a servo control system and a data acquisition and processing system. A vanadium oxide uncooled infrared focal plane detector produced by IRay Technology Co., Ltd. (Shanghai, China) is used in the multispectral infrared imaging gas detection system. The resolution is 640 × 512, and the pixel spacing is 17 μm. The maximum frame rate of the detector is 50 Hz and the NETD is 50 mK@25 °C/F#1. The range of the response is 3 μm~15 μm. The working wavelength of the imaging optical system is 6.5 μm~15 μm, the field of view is 12° × 10°, and the F# is 1.

The filter wheel is composed of six filters, and the operating wavelength of the filters is shown in Table 1. The filter wheel rotates at a constant speed, and the rotating speed is one circle per second. The sampling frequency of the infrared focal plane detector is 30 Hz. The homemade main control circuit sends a trigger signal to the computer software every time the filter wheel rotates a circle. The computer software starts to collect images after receiving the trigger signal, so as to realize the synchronous collection of images in each circle. The background is the ordinary background of the scene. The background emits infrared radiation and there is no additional light source. Hard backgrounds such as concrete floors and walls are helpful for gas detection. Gas detection is difficult when grass and trees are used as background.

### 2.2. Gas Detection Setups

**Figure 2:**
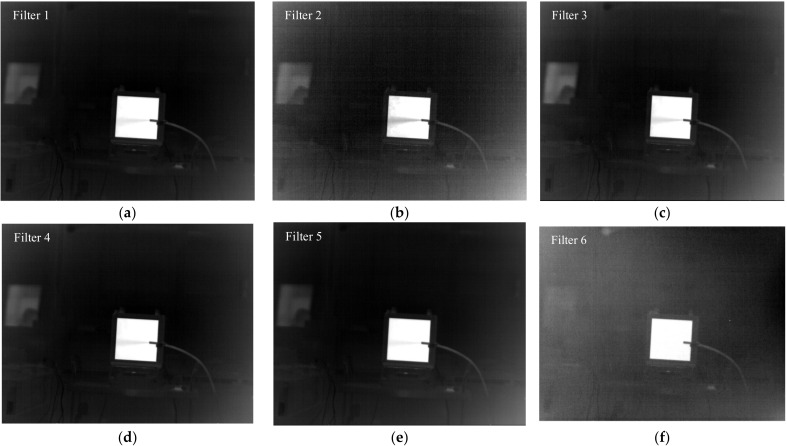
SF_6_ images in front of the blackbody passing through different filters. (**a**) passing through filter 1; (**b**) passing through filter 2; (**c**) passing through filter 3; (**d**) passing through filter 4; (**e**) passing through filter 5; and (**f**) passing through filter 6.

An extended area blackbody produced by CI Systems was used. The temperature of the blackbody was set to be 5 K higher than the ambient temperature. At a room temperature of about 20 °C, SF_6_ was released in front of the blackbody. SF_6_ gas was placed in high-pressure cylinders with a concentration of 100% and released directly through a pressure reducing valve. The images collected by the detector passing through six filters are shown in Figure 2. The gas can be clearly seen in the images passing through Filter 1, Filter 2, Filter 3, Filter 4 and Filter 5, but cannot be seen in the images passing through Filter 6. This corresponds to the band setting of the filters.

**Figure 3:**
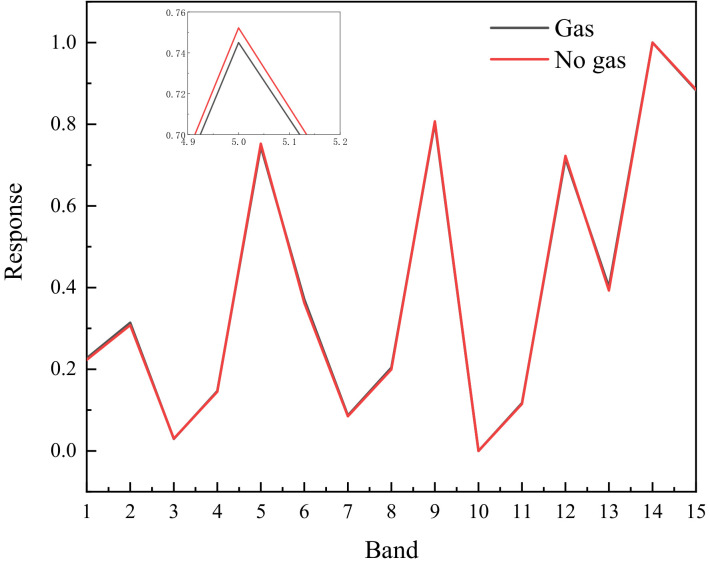
Response of gas region and gas free region in multiple wavebands.

The response in the gas area and the gas free area is presented in Figure 3 after subtracting the six images in pairs. There is no significant difference between the response of the area with gas and without gas in front of the blackbody. The radiation energy of the blackbody is powerful. Although SF_6_ gas can absorb some radiation energy, the detector can still receive a significant amount of background radiation after passing through the gas cloud. It is difficult to distinguish gas based on its response curve in each band. The gas can be clearly seen in one of the differential images, and the gas area can be recognized through image processing. However, this method can only recognize that the gas has an absorption peak in this band. Through the response curve of gas in each band, the gas response of multiple bands can be analyzed to distinguish different types of gas.

**Figure 4:**
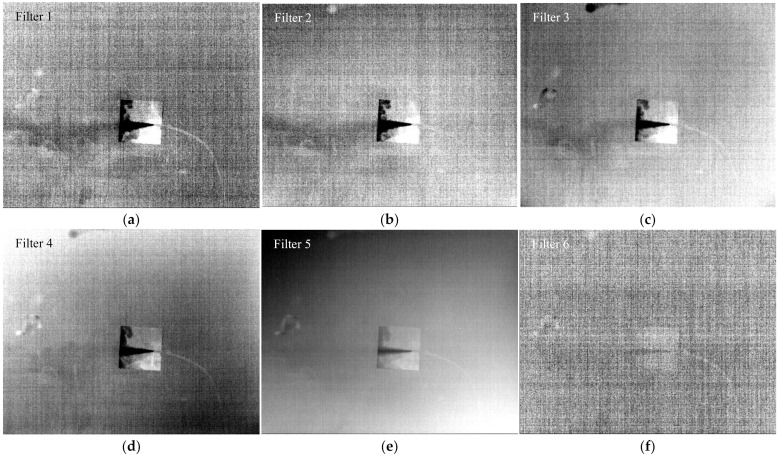
SF_6_ images in front of blackbody passing through different filters after subtracting background. (**a**) passing through Filter 1; (**b**) passing through Filter 2; (**c**) passing through Filter 3; (**d**) passing through Filter 4; (**e**) passing through Filter 5; (**f**) passing through Filter 6.

In order to reduce the influence of background on the response of gas in each band, we subtracted the background from the collected image. We then averaged the previous eight frames as the background of the current image and subtracted the background before image difference. The images of SF_6_ gas before the blackbody and minus the background are shown in Figure 4. After subtracting the background, SF_6_ gas are more noticeable in the images.

**Figure 5:**
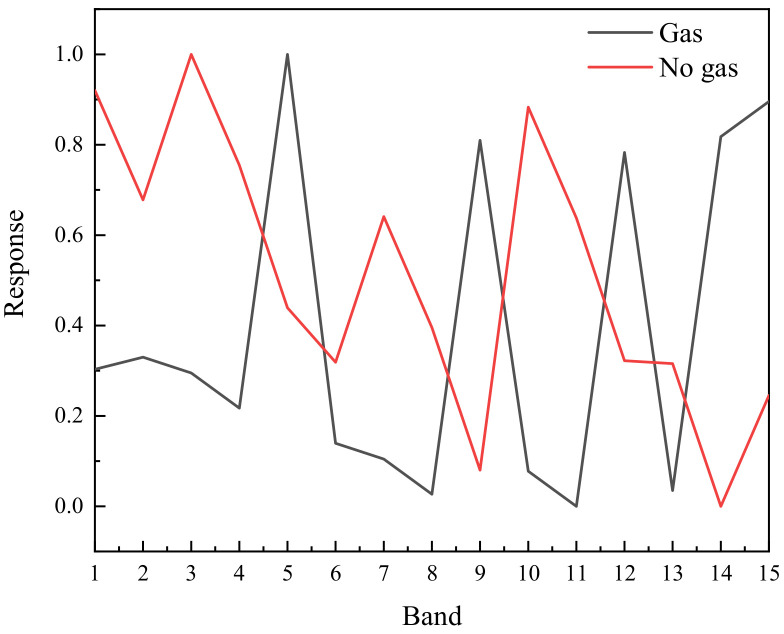
Response of gas region and gas-free region in multiple wavebands after subtracting background.

The six images after removing the background are subtracted in pairs to obtain 15 images. The response curves of gas area and gas-free area are shown in Figure 5. The two curves are clearly distinct, and the gas region is easily identified. The gas region has a high response in the 5th, 9th, 12th, 14th and 15th frame differential images, which is consistent with the theoretical analysis.

### 2.3. Data Processing

**Figure 6:**
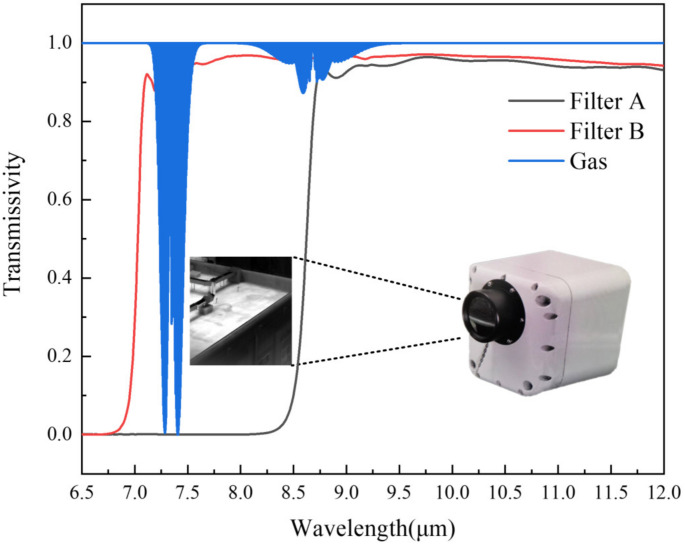
Principle of gas detection by multispectral infrared imaging.

Infrared imaging may not show the gas cloud when its temperature is near to the background. As shown in Figure 6, a pair of filters is added to the multispectral infrared imaging gas detection system in front of the camera. The filters transmit or cut off the infrared absorption peak of the gas, so that the gas in the field of view can be distinguished without considering the temperature of gas and background. The type of gas can be identified by judging the absorption peak of the gas through the filters.

The background and gas cloud are simplified as a two-layer radiative transfer model. Ignoring the influence of atmospheric transmission, the background radiation entering the infrared focal plane detector after passing through the atmosphere and filter is [19].

(1) $$\begin{array}{ccc} L & = & \int \left[L_{BB}(T_{B})\tau_{C}+L_{BB}(T_{C})(1-\tau_{C})\right]\tau_{F}d\lambda \\ & = & \int \left\{\left[L_{BB}(T_{B})-L_{BB}(T_{C})\right]\tau_{C}+L_{BB}(T_{C})\right\}\tau_{F}d\lambda \end{array}$$

where $L_{BB}(T)$ is the spectral luminance of a blackbody at temperature *T*, $T_{B}$ and $T_{C}$ are the temperatures of the background and the gas cloud, respectively. $\tau_{C}$ and $\tau_{F}$ are the transmittance of the gas cloud and filter.

If the temperature of the gas and the background are the same, that is $T_{B}=T_{C}$, the spectral radiation received by the focal plane detector is:

(2) $$L=\int L_{BB}(T_{C})\tau_{F}d\lambda$$

Therefore, even if the gas temperature and background temperature are the same, since the filters have different transmissivity to the gas cloud, the infrared focal plane detector can obtain different spectral radiation energies. The properties of gas cloud can be obtained by difference between two infrared images.

The detector continuously gathers images while the filter wheel rotates at a steady speed. The picture captured by the detector will be partially obstructed when the optical axis of the filter diverges from the optical path. As a result, only six frames of pictures captured by the detector are valid when the filter wheel rotates for one circle. Effective images are located at a fixed number of frames in all collected images, so that they can be selected. The detector outputs 14 bits of raw data, and image processing is used to remove image blindness. After non-uniformity correction, the responsivity of each pixel of the detector is basically consistent. The data collection and processing system captures, analyzes, and saves infrared images, and outputs identification results.

**Figure 7:**
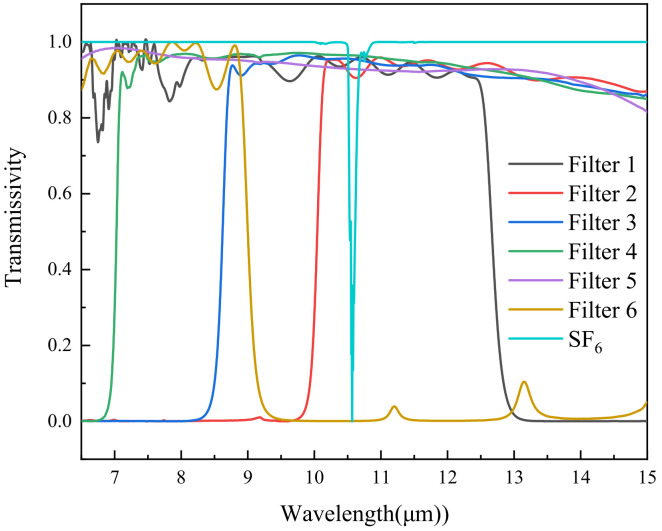
Transmission spectrum of filters and SF_6_.

The spectral system consists of two short-pass filters, three long-pass filters, and one broadband filter. The light transmission bands of the filters are shown in Figure 7. According to HITRAN database, at one atmospheric pressure and a temperature of 298.15 K, the absorption peak of SF_6_ is 10.57 μm. The light at the absorption peak of SF_6_ gas does not pass through the Filter 6, but passes through the other five filters. The detector collects six valid images while the filter wheel rotates one circle. A total of 15 differential images could be obtained by subtracting six images, and the bands that can be detected are shown in Table 2. SF_6_ can be detected in the 5th, 9th, 12th, 14th and 15th differential images, respectively.

The data type output by the camera can be selected as raw data and non-uniformly corrected data. The non-uniformly corrected data eliminate the detector blind element and the image contrast is obvious. Non-uniformly corrected data were selected and then performed a single point correction by placing a baffle between the filters and the lens. To improve the contrast of the gas cloud, we subtracted the background image when there was no gas. Then, we subtracted the image by pairs. The response of the gas in all differential bands was focused on. If the gas has multiple absorption peaks, all of them will be detected. The correlation coefficient between the response of each pixel in the 15-frame differential image and the standard spectral line were calculated. The threshold value of the correlation coefficient is set to 0.9; if it is greater than 0.9, we think it is gas, and if it is less than 0.9, we think it is not.

## 3. Results and Discussion

### 3.1. Validation of the Method in Laboratory Conditions

**Figure 8:**
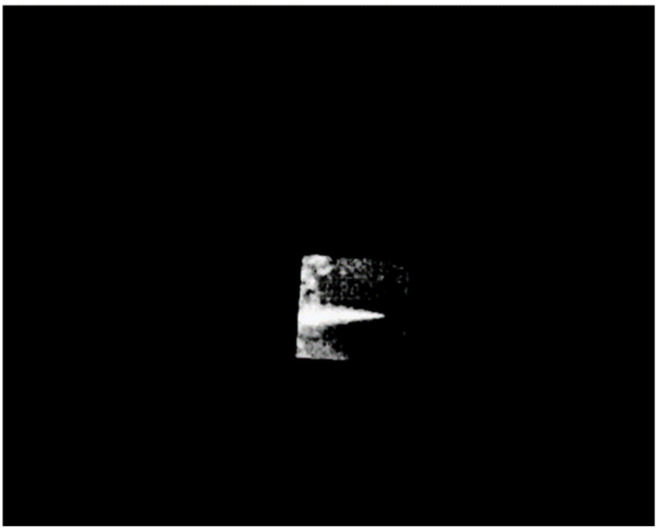
Identified diffusion area of SF_6_ gas.

Assume that the response curve of the gas area in Figure 5 is the standard response curve of SF_6_. The gas was recognized using the standard response curve. The six images after subtracting the background are subtracted in pairs to obtain15 images. The response of each pixel in these 15 frames of images is correlated with the standard spectral line. The gas diffusion area is the pixel with a high correlation coefficient. After threshold segmentation, it is possible to extract the gas diffusion zone [20]. As shown in Figure 8, the diffusion region of SF_6_ gas in front of blackbody can be identified.

**Figure 9:**
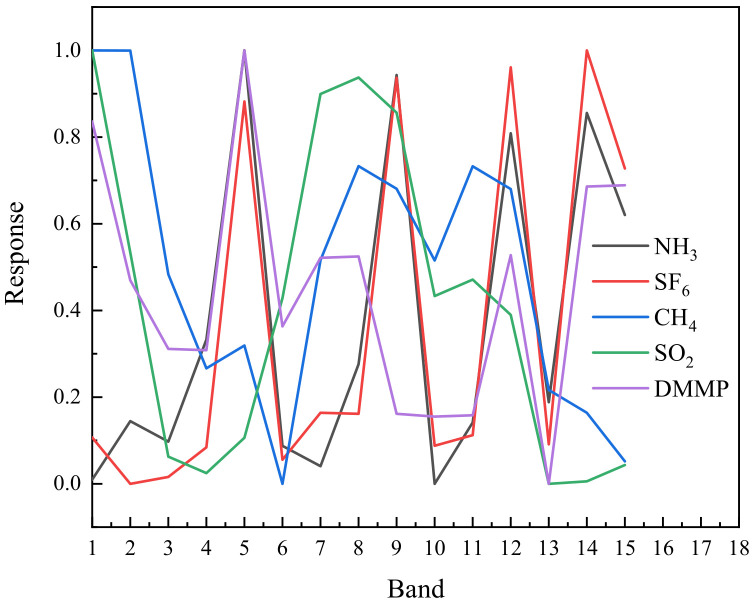
Response spectrum of different gases.

The images of NH_3_, SF_6_, CH_4_, SO_2_ and DMMP were acquired in front of the black body. The response spectrum of each gas can be obtained after subtracting the background, as shown in Figure 9. The main absorption peaks of NH_3_ are 10.35 μm and 10.73 μm. In addition, there are many weak absorption peaks. The absorption peak of SF_6_ is 10.57 μm. There is only one absorption peak. The absorption peaks of NH_3_ and SF_6_ are close, so the multispectral absorption spectra are similar. In the long wave infrared region, CH_4_ has an absorption peak with a wavelength of 7.52 μm. The absorption peak of SO_2_ is 7.32 μm. These two gases have comparable multispectral absorption lines because of their comparable absorption peaks. DMMP has several absorption peaks in the long wave infrared region, which are 7.83 μm, 9.49 μm, 10.87 μm, 12.2 μm, 13.93 μm. Due to its numerous absorption peaks, DMMP stands out from other gases in the absorption spectrum.

### 3.2. Validation in Real-World Conditions

**Figure 10:**
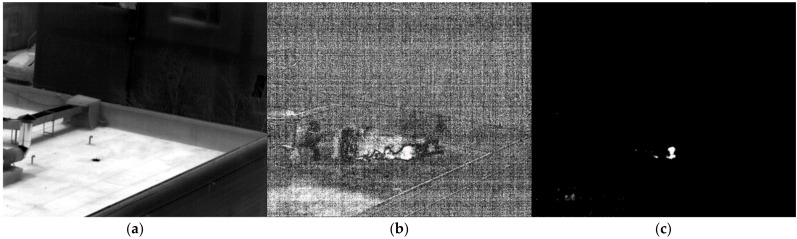
Image of NH_3_ at 48.3 m. (**a**) image passing through filter 1; (**b**) the difference image; (**c**) the detection result.

The multispectral absorption spectrum gathered before the blackbody is used as the reference spectrum to determine the gas. Gas was released on the roof during a sunny day. A laser range finder found the distance to be 48.3 m. Ammonia gas freely evaporated when 200 mL of ammonia water with a 25% concentration was put onto the roof. Figure 10a shows the image passing through filter 1. Figure 10b shows the fifth difference image after subtracting the background. The gas dispersion is clearly visible. Figure 10c displays the results of the detected ammonia following the relevant calculation of the multispectral standard spectral line. It was successfully detected where the ammonia gas that has been volatilized gathers.

**Figure 11:**
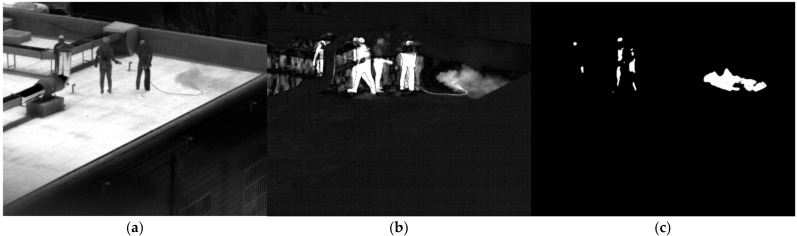
Image of SF_6_ at 48.3 m. (**a**) Image passing through filter 1; (**b**) the difference image; (**c**) the detection result.

The SF_6_ gas in the cylinder was directly released. Figure 11a shows the image of SF6 gas passing through filter 1. After subtracting the background, the ninth difference image is shown in Figure 11b. Humans are clearly seen in the difference image after the background has been removed due to the movement of the people. As shown in Figure 11c, the area of gas can be derived following the comparison of the multispectral standard spectrum. The diffusion area of the gas is accurately identified. However, some of the region in which people move is also misinterpreted for gas. When there is a moving target in the field of view, averaging multiple images as the background can suppress the moving target. However, there will still be moving targets in the background because of the low average number. After removing the background, the moving target is clear. Additionally, the filter wheel is revolving, and the time of image acquisition through each filter is different. The moving objects have different positions on each image, and there will also be moving areas after subtracting. Moving targets might be misinterpreted for gas after the multispectral similarity computation of gas. In order to decrease false alarms, we should try to keep moving targets out of the field of view when identifying gas.

**Figure 12:**
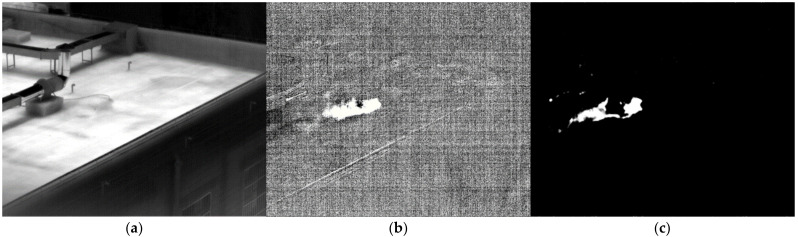
Image of SO_2_ at 48.3 m. (**a**) Image passing through filter 1; (**b**) the difference image; (**c**) the detection result.

The SO_2_ gas in the high-pressure cylinder was directly released. Figure 12a shows the image of SO_2_ gas passing through Filter 1. The area of gas is not obvious. After subtracting the background, the first image after difference is shown in Figure 12b. The diffusion area of gas can be clearly seen. After the similarity calculation of the standard response spectrum of SO_2_ gas, the area of identified gas is shown in Figure 12c. The recognition result is relatively accurate.

**Figure 13:**
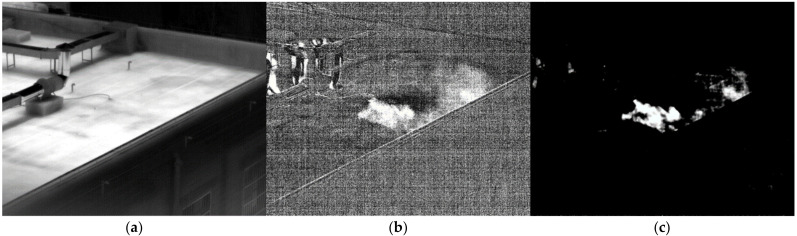
Image of CH_4_ at 48.3 m. (**a**) Image passing through filter 1; (**b**) the difference image; (**c**) the detection result.

The CH_4_ in the high-pressure cylinder was directly released. Figure 13a shows the image of CH_4_ gas passing through Filter 1. Gas is barely visible on the image. After subtracting the background, the first image after difference is shown in Figure 13b. The diffusion area of gas can be clearly seen on the image. Since there are moving people in the background, the image of people appears after subtracting the background. After the similarity calculation of the standard response spectrum of CH_4_ gas, the region of identified gas is shown in Figure 13c. Since there are no moving people in the current image, there are basically no false positives in the identification results.

**Figure 14:**
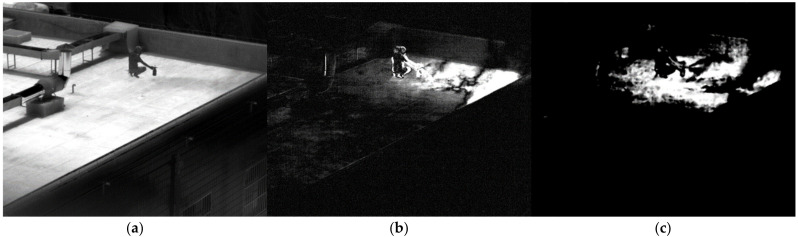
Image of DMMP at 48.3 m. (**a**) Image passing through filter 1; (**b**) the difference image; (**c**) the detection result.

DMMP is liquid at room temperature. A liquid cloud could be created with a sprayer. After spraying, DMMP fell to the ground. Figure 14a shows the picture of DMMP after passing through Filter 1. It is evident from the image that there are moist parts on the ground. After subtracting the background, the first image after difference is shown in Figure 14b. It is easy to see the diffusion area of DMMP. After calculating the similarity of DMMP standard response spectrum, the area of identified gas is shown in Figure 14c. DMMP scattered on the ground can also be identified. There is no false alarm because the individual in the image is not moving.

**Figure 15:**
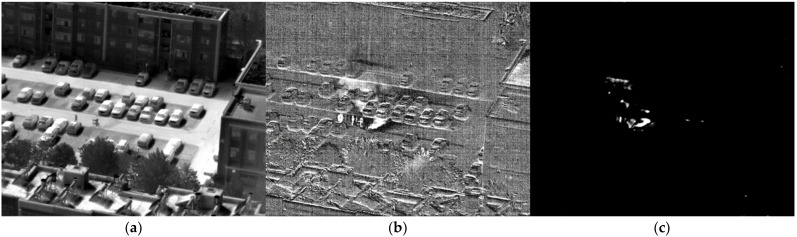
Image of SF_6_ at 252.6 m. (**a**) Image passing through filter 1; (**b**) the difference image; (**c**) the detection result.

At a distance of 252.6 m, SF_6_ gas in the cylinder was sprayed. Figure 15a shows the image of SF6 gas passing through Filter 1. Some gas clouds can be seen in the image. After subtracting the background, the ninth difference image is shown in Figure 15b. The gas cloud and diffusion area can be clearly seen. After the similarity calculation of multispectral standard spectrum, the gas area can be obtained, as shown in Figure 15c. The region where the gas sprayed is precisely located. Probably due to the low gas concentration, only a part of the diffusion area of the gas has been identified.

**Figure 16:**
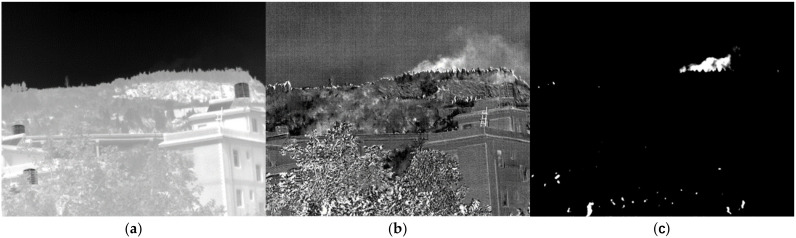
Image of NH_3_ at 1124.5 m. (**a**) Image passing through filter 1; (**b**) the difference image; (**c**) the detection result.

Ammonia water with a concentration of 25% was sprayed with four sprayers on a mountain 1124.5 m from the observation position. The sprayer has a capacity of 15 L and a mist output of 120 L~140 L. As a result, the concentration of ammonia in the sprayed gas cloud was about 3%. The spraying was finished in about 3 min by adding 8 L of ammonia water to each sprayer. As shown in Figure 16a, the image passing through Filter 1 reveals that the gas cloud is not obvious. After subtracting the background, the fifth difference image is shown in Figure 16b. The gas clouds drifting above the mountain are readily visible. After response spectrum similarity detection, the detected gas cloud is shown in Figure 16c. The gas cloud is accurately identified. The ability to see the far-off spreading clouds increases with a decrease in the threshold value. Lowering the threshold, on the other hand, will increase false positives. Some false positives are created by the shaking trees.

## 4. Conclusions

Infrared imaging gas detection technology takes images of the infrared radiation absorbed by the gas and uses image processing to determine the type of gas. The multispectral infrared imaging gas detection system separates infrared radiation, using filters to produce images of different spectral radiations on the infrared spectrum. It could detect gases that have infrared absorption peaks between 6.5 μm and 15 μm. Differential processing was used on infrared multispectral images. The standard absorption spectrum of the multispectral system was determined by collecting the gas in front of a blackbody. The influence of the background was reduced by subtracting the background. It is possible to monitor NH_3_, SF_6_, CH_4_, SO_2_ and DMMP dynamically in real-time. The multispectral infrared imaging gas detection system is capable of long-distance gas detection at distances ranging from tens of meters to one kilometer. In the future, the concentration of the telemetry gas can also be calculated after calibrating the gas concentration in the gas chamber.

## Figures and Tables

**Table 1:** The operating wavelength of the filters.

Number	Operating Wavelength
1	6.5 μm~12.5 μm
2	10 μm~15 μm
3	8.5 μm~15 μm
4	7 μm~15 μm
5	6.5 μm~15 μm
6	6.5 μm~9 μm

**Table 2:** Waveband that can be detected after image subtraction of different filters.

Number	Sequence of Image Subtraction	Detection Range
1	1–2	6.5 μm~10 μm; 12.5 μm~15 μm
2	1–3	6.5 μm~8.5 μm; 12.5 μm~15 μm
3	1–4	6.5 μm~7 μm; 12.5 μm~15 μm
4	1–5	12.5 μm~15 μm
5	1–6	9 μm~12.5 μm
6	2–3	8.5 μm~10 μm
7	2–4	7 μm~10 μm
8	2–5	6.5 μm~10 μm
9	2–6	6.5 μm~9 μm; 10 μm~15 μm
10	3–4	7 μm~8.5 μm
11	3–5	6.5 μm~8.5 μm
12	3–6	6.5 μm~8.5 μm; 9 μm~15 μm
13	4–5	6.5 μm~7 μm
14	4–6	6.5 μm~7 μm; 9 μm~15 μm
15	5–6	9 μm~15 μm

## Data Availability

Not applicable.
